# Analyzing the effect of laparoscopy duration time on peroperative gastroesophageal reflux

**DOI:** 10.3906/sag-1803-176

**Published:** 2019-04-18

**Authors:** Gökhan Berktuğ BAHADIR, Hakan TAŞKINLAR, Caner İSBİR, İsa KILLI, Dilek YÜNLÜEL, Ülkü ÇÖMELEKOĞLU, Ali NAYCI

**Affiliations:** 1 Department of Pediatric Surgery, Faculty of Medicine, Mersin University, Mersin Turkey; 2 Department of Biophysics, Faculty of Medicine, Mersin University, Mersin Turkey

**Keywords:** Gastroesophageal reflux, laparoscopy, rat model

## Abstract

**Background/aim:**

Intraabdominal pressure (IAP) is one of the main reasons for gastroesophageal reflux (GER). This study investigates whether IAP during laparoscopic surgery leads to GER in a time-dependent manner.

**Materials and methods:**

In a laparoscopy model, 15 mmHg IAP was created in 8 Wistar albino rats in the Trendelenburg position (TP). A 5 mm laparotomy was performed in the left lower abdominal region, and a 6 Fr catheter was placed intraabdominally. Air was insufflated into the abdominal cavity, and the pressure was kept constant at 15 mmHg. Esophageal pH alterations were measured by pH sticks for 4 h every 30 min.

**Results:**

The basal median esophageal pH value was 9 (8–10), the value after placing the catheter was 9 (7–10) (P = 0.47), and the median pH value after placing the subjects in TP was 9 (8–10) (P = 0.70). In our experimental model, esophageal pH values were found to decrease significantly at the 150th minute in TP and at 15 mmHg IAP (P < 0.05). Two rats died: one at the 120th minute and the other at the 240th minute (P > 0.05).

**Conclusion:**

Esophageal pH values decreased and continued to remain low following IAP increase and TP in this experimental rat model. Prolonged laparoscopic procedures can particularly lead to GER that requires instant recognition and rapid and appropriate intervention.

## 1. Introduction

Gastroesophageal reflux (GER) is the regurgitation of gastric contents into the esophagus. Acidic gastric contents have detrimental effects in the esophagus, pharynx, and airways, which normally contain alkali media. Children are more prone to have GER than adults. However, with the maturation of the antireflux mechanisms, the prevalence of GER dramatically decreases by adulthood [1]. GER causes various disorders including esophagitis, otitis, sinusitis, reactive airway disease, and aspiration that leads to pneumonia [2]. Increased intraabdominal pressure (IAP) is one of the main mechanisms that lead to GER. Moreover, obesity, ascites, and peritoneal dialysis are also known to cause GER [3–5]. GER is also encountered after the closure of anterior abdominal wall defects such as gastroschisis and omphalocele [6,7].

Laparoscopic surgery is the preferred method in many surgical procedures in many centers nowadays, due to its known advantages over open surgery. Theoretically, intraabdominal CO2 insufflation and the Trendelenburg position (TP) trigger GER by increasing IAP during laparoscopic surgery. Additionally, GER is also promoted by the lack of a fasting period in urgent laparoscopic procedures and the disruption of mechanisms such as gravity, swallow reflex, salivation, and esophageal motility, which protect the esophagus from gastric acid under general anesthesia [8]. Tracheal aspiration of gastric content during laparoscopic surgery is a life-threatening complication that requires immediate airway management [9,10]. This study aimed to analyze the effect of IAP and Trendelenburg positioning on gastroesophageal reflux in a time-dependent manner.

## 2. Materials and methods

This study was approved by the university’s Animal Studies Local Ethics Committee on 26 April 2016 (No. 52602694-050/e98165). The study group included 8 Wistar albino rats with a mean weight of 232 (180–300) g. According to the reduction and refinement principles of animal research and the statistically significant sample model, the number of rats was reduced to the minimum number in this experiment. The rats were kept in galvanized cages in 12-h light/12-h dark cycle at an ambient temperature of 22 °C and humidity of 50%. They were fed on standard rat pellet and given ad libitum access to water. They were fasted overnight prior to the procedure and anesthetized with 80 mg/kg ketamine HCl (Alfamine 10%, İzmir, Turkey) and 7 mg/kg xylazine (Xylazinbio 2%, Bioveta, Czech Republic) administered via intraperitoneal route.

**Figure 1:**
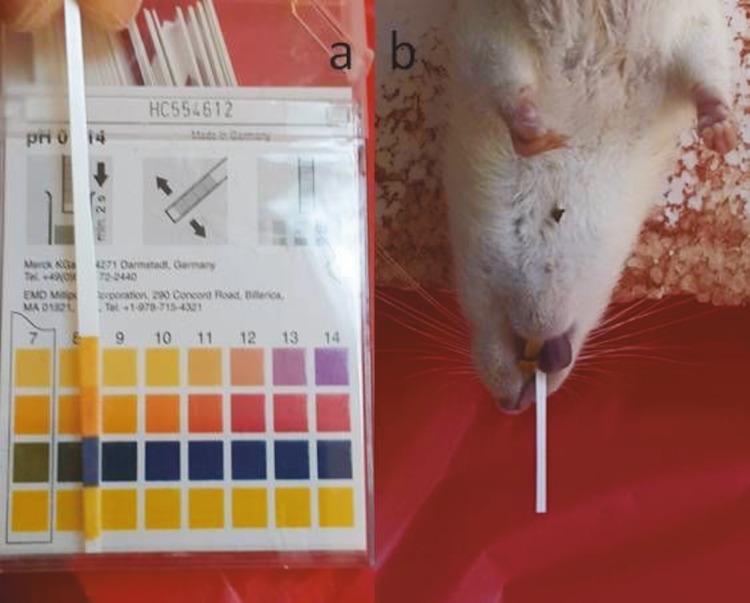
pH-strip (a) and oral application in a rat (b).

After induction of anesthesia, the rats were prepared in supine position and a pH-strip with 2 mm thickness (pH-indicator strips, MColorplast, Merck, Germany) was introduced 3 cm from the oral opening through the anterior teeth into the esophagus to record pH values (Figures 1a and 1b). A 5 mm laparotomy was performed from the left lower abdominal region, and a 6 Fr catheter was placed intraabdominally. Air-tightness was provided by placing a circular suture using 3/0 silk suture (Doğsan, Turkey). After the procedure, a new pH-strip was placed in the same manner in order to obtain the new measurement. The rats were placed at 30° TP, and the esophageal pH measurement was repeated. IAP alterations were measured instantly using the BIOPAC MP 100 electrophysiological recording station (Santa Barbara, CA, USA). The intraabdominal catheter was attached to the pressure converter and GTA 200 amplifier. The pressure data were transferred to a computer with a 16 byte digital converter at a sample speed of 200 s. The values prior to air insufflation were noted as the basal values. Air was insufflated into the abdominal cavity, and the pressure was kept constant at 15 mmHg. Esophageal pH measurements were repeated every 30 min for 240 min (Table 1). Color changes on the pH-strip were evaluated by 2 observers according to the reference values (Table 2).

**Table 1:** Measurements of esophageal PH values.

	pH values								Rat 1	Rat 2	Rat 3	Rat 4	Rat 5	Rat 6	Rat 7	Rat 8
	During anesthesia	9	8	9	9	10	10	9	9
During inserting catheter with minilaparotomy	7	8	9	8	9	9	9	10
In Trendelenburg position	9	10	10	8	9	8	9	9
30th minute	9	10	7	8	9	9	9	9
60th minute	9	9	7	5	9	8	9	8
90th minute	10	8	7	5	8	8	8	8
120th minute	10	9	5	5	Exitus	7	9	6
150th minute	9	8	4	4		7	7	7
180th minute	6	6	5	5		7	7	6
210th minute	4	4	4	5		6	6	6
240th minute	4	5	5	Exitus		7	7	7

**Table 2:** Esophageal pH changes between 0 and 240 min under application of intraabdominal pressure and Trendelenburg position; a drop in pH was observed after the 150th minute (a statistically significant decrease in the pH value was observed after the 150th minute).

Preprocedural pH				Postprocedural pH				30° Trendelenburg pH			
Median 9 (8–10)				Median 9 (7–10); P = 0.47				Median 9 (8–10); P = 0.70			
15 mmHg intraabdominal pressure and 30° Trendelenburg position											
Minute	0th	30th	60th		90th	120th	150th		180th	210th	240th
pH	9(8–10)	9(5–9)	8(5–10)		8(5–10)	7(4–9)	6(4–7)		5(4–7)	5(4–6)	7(5–7)
P	1.00	0.20	0.24		0.14	0.07	0.005		0.003	0.001	0.003

The study data were analyzed using repeated measures analysis in SPSS 17. A value of less than 0.05 was considered statistically significant.

## 3. Results

The basal median esophageal pH value was 9 (8–10), the value after placing the catheter was 9 (7–10) (P = 0.47), and the median pH value after placing the subjects in TP was 9 (8–10) (P = 0.70). In our experimental model, esophageal pH values were found to have decreased significantly at the 150th minute in TP and at 15 mmHg IAP (P < 0.05) (Table 3). Two rats died: one at the 120th minute and the other at the 240th minute (P > 0.05). There were no macroscopic pathological findings in the thoracic and abdominal compartments in either rat. There were also no histological findings in the trachea, lungs, and esophagus at their autopsy.

## 4. Discussion

In this experimental animal model, esophageal pH value decreased significantly after the 150th minute and remained low throughout the study at 15 mmHg IAP and in TP.

Laparoscopic surgery is a method preferred over open procedures for antireflux surgery nowadays. However, it is not clearly identified that laparoscopic surgery may directly contribute to GER. In order to create a working space in laparoscopic surgical procedures, carbon dioxide (CO2) is insufflated into the peritoneal cavity at a pressure of 12–15 mmHg and a rate of 3–5 L/min according to the size and age of the patient. This level of pressure is maintained throughout the procedure. In order to remove bowels from the operative field, particularly during pelvic operations such as acute appendicitis, ovarian cyst, undescended testis, and recto-vesical fistula, patients are placed in TP. This pressure and position change is presumed to affect esophagus-stomach anatomy and function [1]. Doyle et al. reported a rate of 47% for GER during laparoscopic cholecystectomy procedures and 15% during laparoscopic gynecological procedures [11]. Interestingly, our study showed no esophageal pH change associated with pressure and position changes within the first 2 h. Unlike the literature data, we evaluated the effect of IAP and TP on esophageal pH in a specific period of time and noted that esophageal pH dropped after the 150th minute. As it is beyond the scope of our study, we are unable to explain why it dropped after a certain time point. İmamoğlu et al., in an experimental study, observed that IAP reduced testicular blood flow, but it was similar at the 10th and 50th minutes [12]. However, they did not make any measurements for more than 1 h. Further studies are needed to clarify these points.

Dodds et al. showed that abdominal pressure increases with the Valsalva maneuver or drawing the knees and legs to the abdomen, which directly affected intragastric and/or lower esophageal pressure [13]. Iwakiri et al. reported that body position affected lower esophageal pressure in supine and upright positions [14]. Tournadre et al. reported that a 15 mmHg IAP and TP increased lower esophageal sphincter and barrier pressures. They did not detect any cases of GER, but they did detect 2 patients with low pressure levels (18%) [15]. However, some other studies provided conflicting results and interpretations for the subject. Derakhsan et al. performed 24-h pH-meter monitorization and upper gastrointestinal system manometry in obese patients. The authors noted that increased IP affected lower gastroesophageal barrier functions unfavorably and acid reflux occurred into the esophagus [16]. There was no change in GER with positioning in this experiment.

The limitation of this study was a reduced number of subjects to follow the refinement protocol of animal ethics, and also performing esophageal pH measurement with pH-strips using a categorical method and taking only cross-sectional measurements. Another limitation was the absence of alkaline reflux measurement with this technique. Therefore, a study with appropriate animal models, investigating alternations in esophageal pH associated with pressure/position changes and using a 24-h pH-meter monitor and esophagus manometer, would provide further information.

Our study revealed that IAP caused GER over time in an experimental animal model. Long operative time suggests the risk of GER in otherwise brief laparoscopic surgeries such as those for appendicitis, ovarian cysts, or undescended testes, and GER may occur in long-lasting laparoscopic surgeries. This study may provide useful information that families should be given when providing informed consent before surgery. Our findings should be further verified by future studies.
